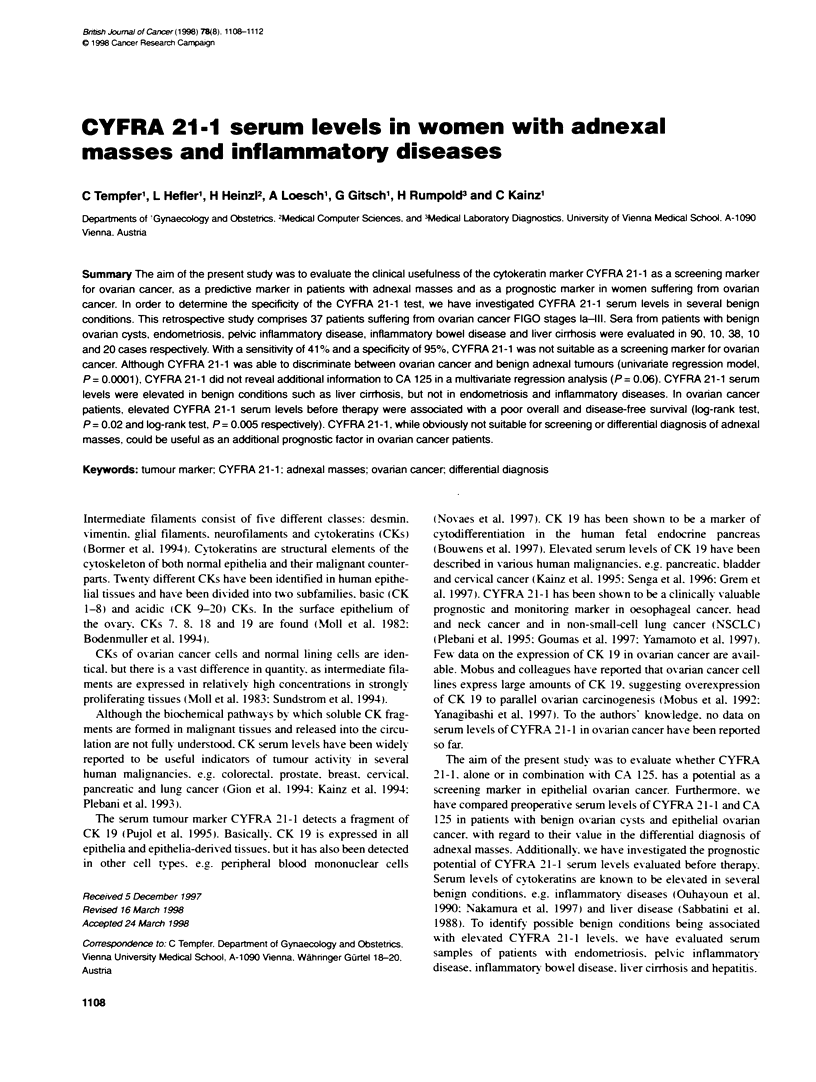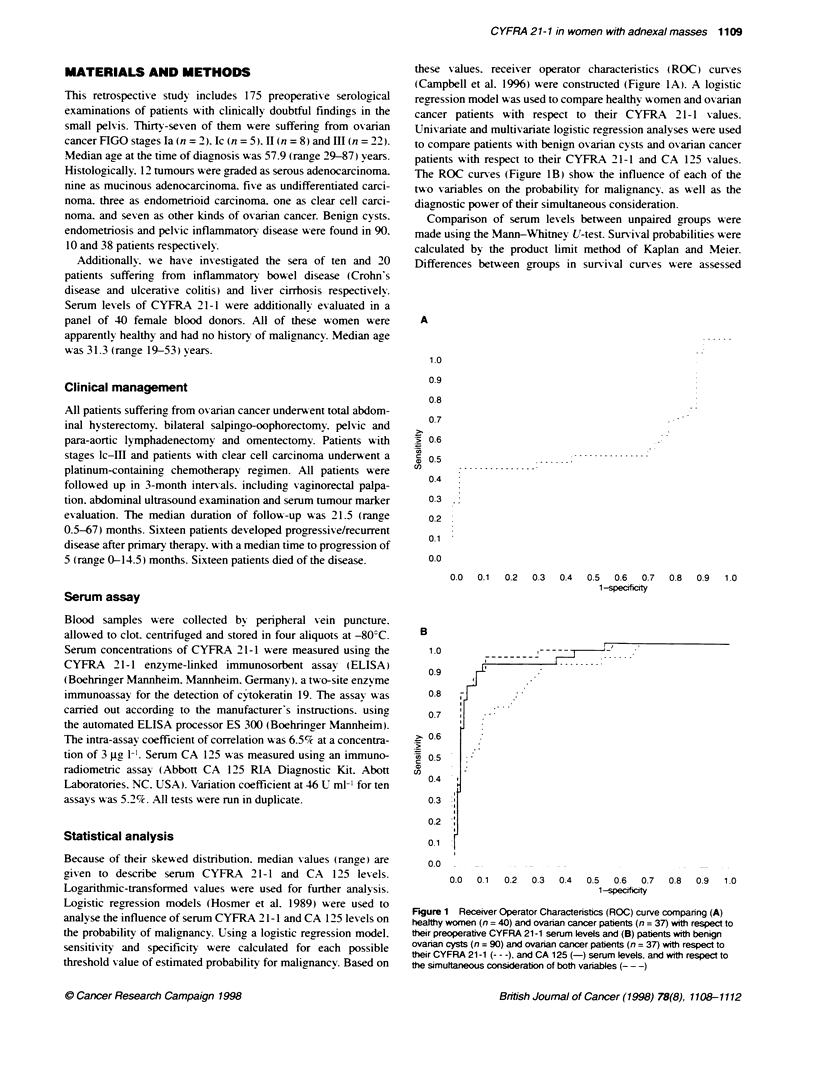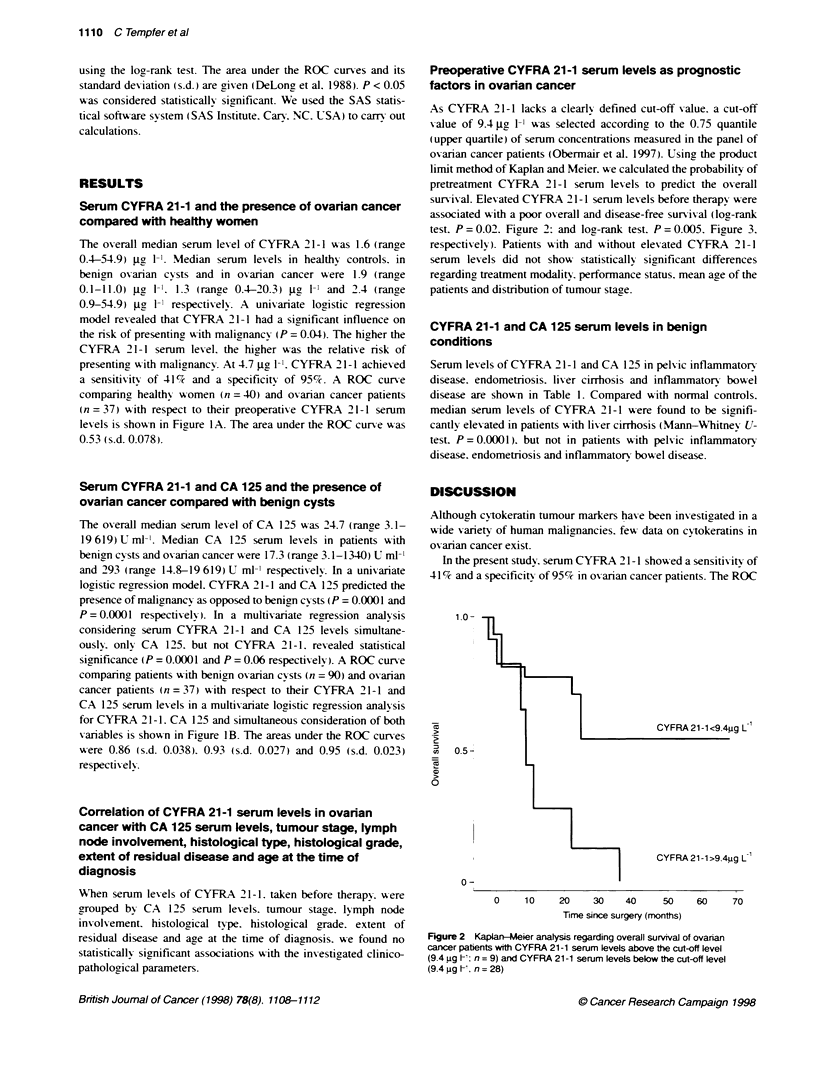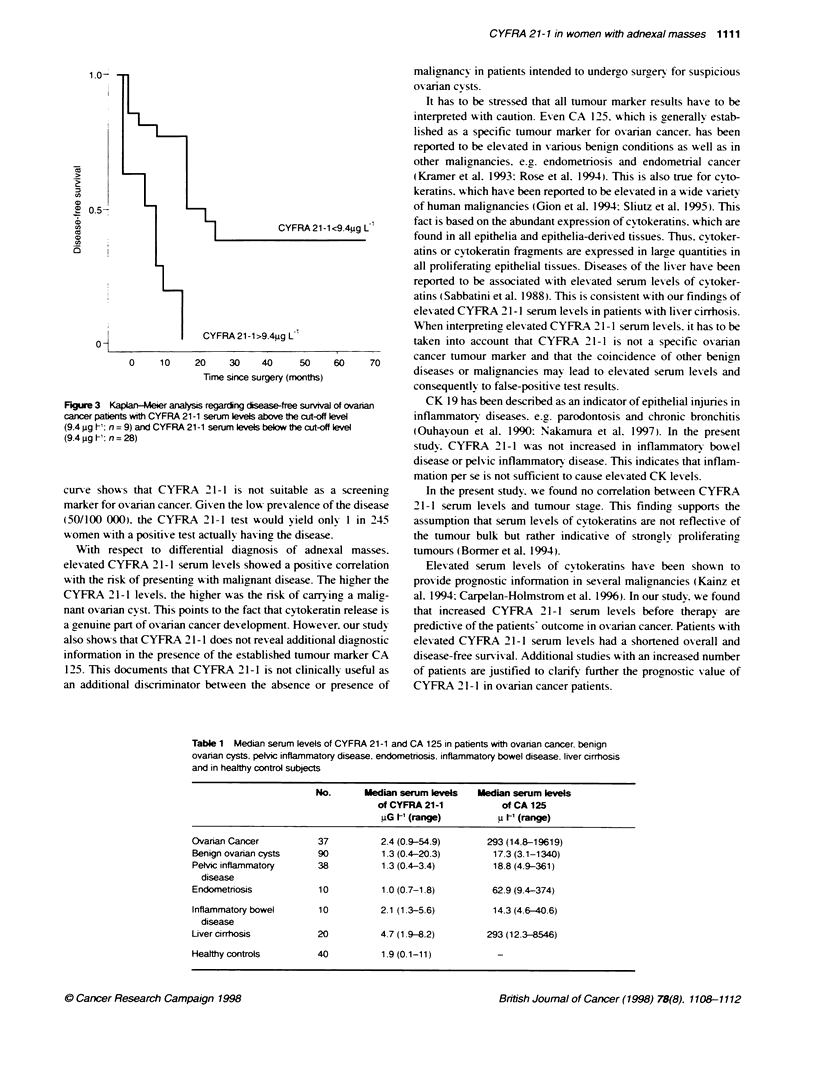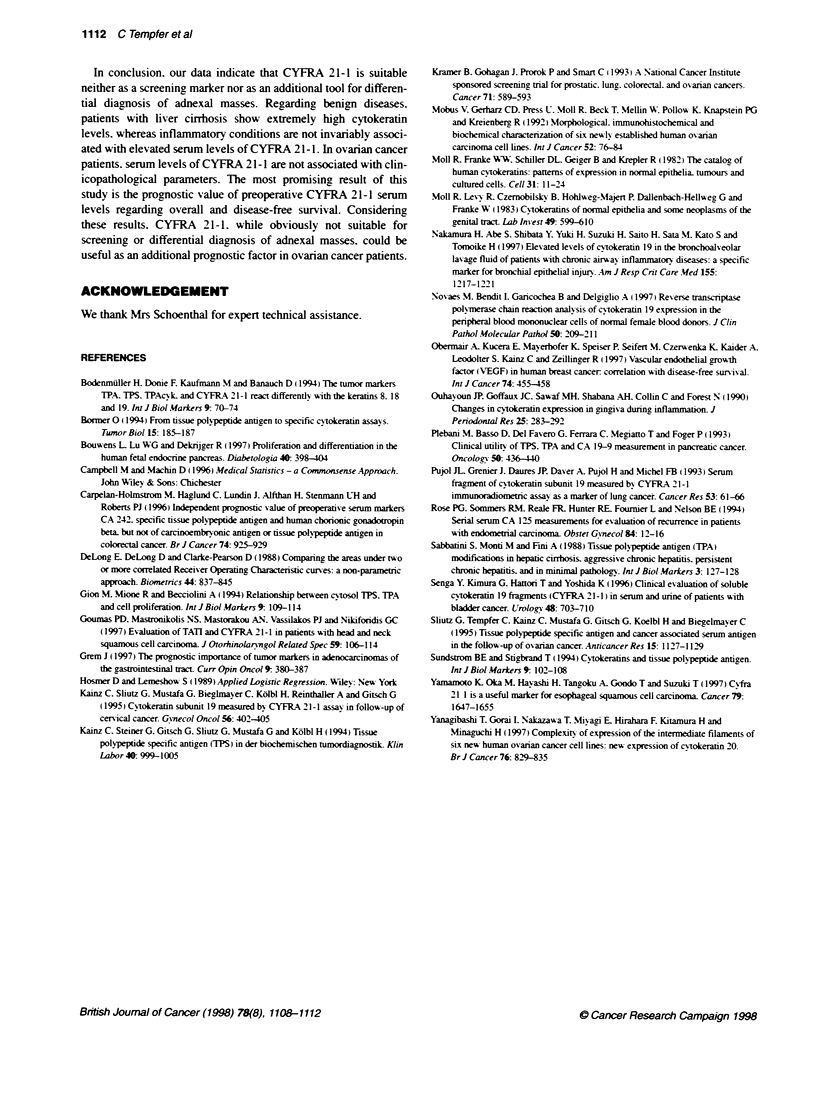# CYFRA 21-1 serum levels in women with adnexal masses and inflammatory diseases.

**DOI:** 10.1038/bjc.1998.636

**Published:** 1998-10

**Authors:** C. Tempfer, L. Hefler, H. Heinzl, A. Loesch, G. Gitsch, H. Rumpold, C. Kainz

**Affiliations:** Department of Gynaecology and Obstetrics, University of Vienna Medical School, Austria.

## Abstract

The aim of the present study was to evaluate the clinical usefulness of the cytokeratin marker CYFRA 21-1 as a screening marker for ovarian cancer, as a predictive marker in patients with adnexal masses and as a prognostic marker in women suffering from ovarian cancer. In order to determine the specificity of the CYFRA 21-1 test, we have investigated CYFRA 21-1 serum levels in several benign conditions. This retrospective study comprises 37 patients suffering from ovarian cancer FIGO stages Ia-III. Sera from patients with benign ovarian cysts, endometriosis, pelvic inflammatory disease, inflammatory bowel disease and liver cirrhosis were evaluated in 90, 10, 38, 10 and 20 cases respectively. With a sensitivity of 41% and a specificity of 95%, CYFRA 21-1 was not suitable as a screening marker for ovarian cancer. Although CYFRA 21-1 was able to discriminate between ovarian cancer and benign adnexal tumours (univariate regression model, P = 0.0001), CYFRA 21-1 did not reveal additional information to CA 125 in a multivariate regression analysis (P = 0.06). CYFRA 21-1 serum levels were elevated in benign conditions such as liver cirrhosis, but not in endometriosis and inflammatory diseases. In ovarian cancer patients, elevated CYFRA 21-1 serum levels before therapy were associated with a poor overall and disease-free survival (log-rank test, P = 0.02 and log-rank test, P = 0.005 respectively). CYFRA 21-1, while obviously not suitable for screening or differential diagnosis of adnexal masses, could be useful as an additional prognostic factor in ovarian cancer patients.


British Journal of Cancer (1998) 78(8). 1108–1112

© 1998 Cancer Research Campaign

### CYFRA 21-1 serum levels in women with adnexal masses and inflammatory diseases

C Tempfer[1], L Hefler[1], H Heinzl[2], A Loesch[1], G Gitsch[1], H Rumpold[3] and C Kainz[1]

Departments of [1]Gynaecology and Obstetrics. [2]Medical Computer Sciences. and [3]Medical Laboratory Diagnostics. University of Vienna Medical School. A-1090 Vienna. Austria

**Summary** The aim of the present study was to evaluate the clinical usefulness of the cytokeratin marker CYFRA 21-1 as a screening marker for ovarian cancer, as a predictive marker in patients with adnexal masses and as a prognostic marker in women suffering from ovarian cancer. In order to determine the specificity of the CYFRA 21-1 test, we have investigated CYFRA 21-1 serum levels in several benign conditions. This retrospective study comprises 37 patients suffering from ovarian cancer FIGO stages Ia–III. Sera from patients with benign ovarian cysts, endometriosis, pelvic inflammatory disease, inflammatory bowel disease and liver cirrhosis were evaluated in 90, 10, 38, 10 and 20 cases respectively. With a sensitivity of 41% and a specificity of 95%, CYFRA 21-1 was not suitable as a screening marker for ovarian cancer. Although CYFRA 21-1 was able to discriminate between ovarian cancer and benign adnexal tumours (univariate regression model, $P = 0.0001$), CYFRA 21-1 did not reveal additional information to CA 125 in a multivariate regression analysis ($P = 0.06$). CYFRA 21-1 serum levels were elevated in benign conditions such as liver cirrhosis, but not in endometriosis and inflammatory diseases. In ovarian cancer patients, elevated CYFRA 21-1 serum levels before therapy were associated with a poor overall and disease-free survival (log-rank test, $P = 0.02$ and log-rank test, $P = 0.005$ respectively). CYFRA 21-1, while obviously not suitable for screening or differential diagnosis of adnexal masses, could be useful as an additional prognostic factor in ovarian cancer patients.

**Keywords:** tumour marker; CYFRA 21-1; adnexal masses; ovarian cancer; differential diagnosis

Intermediate filaments consist of five different classes: desmin. vimentin. glial filaments. neurofilaments and cytokeratins (CKs) (Bormer et al. 1994). Cytokeratins are structural elements of the cytoskeleton of both normal epithelia and their malignant counterparts. Twenty different CKs have been identified in human epithelial tissues and have been divided into two subfamilies. basic (CK 1–8) and acidic (CK 9–20) CKs. In the surface epithelium of the ovary. CKs 7. 8. 18 and 19 are found (Moll et al. 1982: Bodenmuller et al. 1994).

CKs of ovarian cancer cells and normal lining cells are identical. but there is a vast difference in quantity. as intermediate filaments are expressed in relatively high concentrations in strongly proliferating tissues (Moll et al. 1983: Sundstrom et al. 1994).

Although the biochemical pathways by which soluble CK fragments are formed in malignant tissues and released into the circulation are not fully understood. CK serum levels have been widely reported to be useful indicators of tumour activity in several human malignancies. e.g. colorectal. prostate. breast. cervical. pancreatic and lung cancer (Gion et al. 1994: Kainz et al. 1994: Plebani et al. 1993).

The serum tumour marker CYFRA 21-1 detects a fragment of CK 19 (Pujol et al. 1995). Basically. CK 19 is expressed in all epithelia and epithelia-derived tissues. but it has also been detected in other cell types. e.g. peripheral blood mononuclear cells (Novaes et al. 1997). CK 19 has been shown to be a marker of cytodifferentiation in the human fetal endocrine pancreas (Bouwens et al. 1997). Elevated serum levels of CK 19 have been described in various human malignancies. e.g. pancreatic. bladder and cervical cancer (Kainz et al. 1995: Senga et al. 1996: Grem et al. 1997). CYFRA 21-1 has been shown to be a clinically valuable prognostic and monitoring marker in oesophageal cancer. head and neck cancer and in non-small-cell lung cancer (NSCLC) (Plebani et al. 1995: Goumas et al. 1997: Yamamoto et al. 1997). Few data on the expression of CK 19 in ovarian cancer are available. Mobus and colleagues have reported that ovarian cancer cell lines express large amounts of CK 19. suggesting overexpression of CK 19 to parallel ovarian carcinogenesis (Mobus et al. 1992: Yanagibashi et al. 1997). To the authors' knowledge. no data on serum levels of CYFRA 21-1 in ovarian cancer have been reported so far.

The aim of the present study was to evaluate whether CYFRA 21-1. alone or in combination with CA 125. has a potential as a screening marker in epithelial ovarian cancer. Furthermore. we have compared preoperative serum levels of CYFRA 21-1 and CA 125 in patients with benign ovarian cysts and epithelial ovarian cancer. with regard to their value in the differential diagnosis of adnexal masses. Additionally. we have investigated the prognostic potential of CYFRA 21-1 serum levels evaluated before therapy. Serum levels of cytokeratins are known to be elevated in several benign conditions. e.g. inflammatory diseases (Ouhayoun et al. 1990: Nakamura et al. 1997) and liver disease (Sabbatini et al. 1988). To identify possible benign conditions being associated with elevated CYFRA 21-1 levels. we have evaluated serum samples of patients with endometriosis. pelvic inflammatory disease. inflammatory bowel disease. liver cirrhosis and hepatitis.

*Received 5 December 1997*
*Revised 16 March 1998*
*Accepted 24 March 1998*

*Correspondence to:* C Tempfer. Department of Gynaecology and Obstetrics. Vienna University Medical School. A-1090 Vienna. Währinger Gürtel 18–20. Austria

### MATERIALS AND METHODS

This retrospective study includes 175 preoperative serological examinations of patients with clinically doubtful findings in the small pelvis. Thirty-seven of them were suffering from ovarian cancer FIGO stages Ia ($n = 2$), Ic ($n = 5$), II ($n = 8$) and III ($n = 22$). Median age at the time of diagnosis was 57.9 (range 29–87) years. Histologically, 12 tumours were graded as serous adenocarcinoma, nine as mucinous adenocarcinoma, five as undifferentiated carcinoma, three as endometrioid carcinoma, one as clear cell carcinoma, and seven as other kinds of ovarian cancer. Benign cysts, endometriosis and pelvic inflammatory disease were found in 90, 10 and 38 patients respectively.

Additionally, we have investigated the sera of ten and 20 patients suffering from inflammatory bowel disease (Crohn's disease and ulcerative colitis) and liver cirrhosis respectively. Serum levels of CYFRA 21-1 were additionally evaluated in a panel of 40 female blood donors. All of these women were apparently healthy and had no history of malignancy. Median age was 31.3 (range 19–53) years.

### Clinical management

All patients suffering from ovarian cancer underwent total abdominal hysterectomy, bilateral salpingo-oophorectomy, pelvic and para-aortic lymphadenectomy and omentectomy. Patients with stages Ic–III and patients with clear cell carcinoma underwent a platinum-containing chemotherapy regimen. All patients were followed up in 3-month intervals, including vaginorectal palpation, abdominal ultrasound examination and serum tumour marker evaluation. The median duration of follow-up was 21.5 (range 0.5–67) months. Sixteen patients developed progressive/recurrent disease after primary therapy, with a median time to progression of 5 (range 0–14.5) months. Sixteen patients died of the disease.

### Serum assay

Blood samples were collected by peripheral vein puncture, allowed to clot, centrifuged and stored in four aliquots at –80°C. Serum concentrations of CYFRA 21-1 were measured using the CYFRA 21-1 enzyme-linked immunosorbent assay (ELISA) (Boehringer Mannheim, Mannheim, Germany), a two-site enzyme immunoassay for the detection of cytokeratin 19. The assay was carried out according to the manufacturer's instructions, using the automated ELISA processor ES 300 (Boehringer Mannheim). The intra-assay coefficient of correlation was 6.5% at a concentration of 3 $\mu g\ l^{-1}$. Serum CA 125 was measured using an immunoradiometric assay (Abbott CA 125 RIA Diagnostic Kit, Abbott Laboratories, NC, USA). Variation coefficient at 46 U $ml^{-1}$ for ten assays was 5.2%. All tests were run in duplicate.

### Statistical analysis

Because of their skewed distribution, median values (range) are given to describe serum CYFRA 21-1 and CA 125 levels. Logarithmic-transformed values were used for further analysis. Logistic regression models (Hosmer et al, 1989) were used to analyse the influence of serum CYFRA 21-1 and CA 125 levels on the probability of malignancy. Using a logistic regression model, sensitivity and specificity were calculated for each possible threshold value of estimated probability for malignancy. Based on these values, receiver operator characteristics (ROC) curves (Campbell et al, 1996) were constructed (Figure 1A). A logistic regression model was used to compare healthy women and ovarian cancer patients with respect to their CYFRA 21-1 values. Univariate and multivariate logistic regression analyses were used to compare patients with benign ovarian cysts and ovarian cancer patients with respect to their CYFRA 21-1 and CA 125 values. The ROC curves (Figure 1B) show the influence of each of the two variables on the probability for malignancy, as well as the diagnostic power of their simultaneous consideration.

Comparison of serum levels between unpaired groups were made using the Mann–Whitney $U$-test. Survival probabilities were calculated by the product limit method of Kaplan and Meier. Differences between groups in survival curves were assessed

**Figure 1** Receiver Operator Characteristics (ROC) curve comparing (**A**) healthy women ($n = 40$) and ovarian cancer patients ($n = 37$) with respect to their preoperative CYFRA 21-1 serum levels and (**B**) patients with benign ovarian cysts ($n = 90$) and ovarian cancer patients ($n = 37$) with respect to their CYFRA 21-1 (- - -), and CA 125 (—) serum levels, and with respect to the simultaneous consideration of both variables (– – –)

© Cancer Research Campaign 1998

using the log-rank test. The area under the ROC curves and its standard deviation (s.d.) are given (DeLong et al. 1988). $P < 0.05$ was considered statistically significant. We used the SAS statistical software system (SAS Institute, Cary, NC, USA) to carry out calculations.

### RESULTS

### Serum CYFRA 21-1 and the presence of ovarian cancer compared with healthy women

The overall median serum level of CYFRA 21-1 was 1.6 (range 0.4–54.9) µg $l^{-1}$. Median serum levels in healthy controls, in benign ovarian cysts and in ovarian cancer were 1.9 (range 0.1–11.0) µg $l^{-1}$, 1.3 (range 0.4–20.3) µg $l^{-1}$ and 2.4 (range 0.9–54.9) µg $l^{-1}$ respectively. A univariate logistic regression model revealed that CYFRA 21-1 had a significant influence on the risk of presenting with malignancy ($P = 0.04$). The higher the CYFRA 21-1 serum level, the higher was the relative risk of presenting with malignancy. At 4.7 µg $l^{-1}$, CYFRA 21-1 achieved a sensitivity of 41% and a specificity of 95%. A ROC curve comparing healthy women ($n = 40$) and ovarian cancer patients ($n = 37$) with respect to their preoperative CYFRA 21-1 serum levels is shown in Figure 1A. The area under the ROC curve was 0.53 (s.d. 0.078).

### Serum CYFRA 21-1 and CA 125 and the presence of ovarian cancer compared with benign cysts

The overall median serum level of CA 125 was 24.7 (range 3.1–19 619) U $ml^{-1}$. Median CA 125 serum levels in patients with benign cysts and ovarian cancer were 17.3 (range 3.1–1340) U $ml^{-1}$ and 293 (range 14.8–19 619) U $ml^{-1}$ respectively. In a univariate logistic regression model, CYFRA 21-1 and CA 125 predicted the presence of malignancy as opposed to benign cysts ($P = 0.0001$ and $P = 0.0001$ respectively). In a multivariate regression analysis considering serum CYFRA 21-1 and CA 125 levels simultaneously, only CA 125, but not CYFRA 21-1, revealed statistical significance ($P = 0.0001$ and $P = 0.06$ respectively). A ROC curve comparing patients with benign ovarian cysts ($n = 90$) and ovarian cancer patients ($n = 37$) with respect to their CYFRA 21-1 and CA 125 serum levels in a multivariate logistic regression analysis for CYFRA 21-1, CA 125 and simultaneous consideration of both variables is shown in Figure 1B. The areas under the ROC curves were 0.86 (s.d. 0.038), 0.93 (s.d. 0.027) and 0.95 (s.d. 0.023) respectively.

### Correlation of CYFRA 21-1 serum levels in ovarian cancer with CA 125 serum levels, tumour stage, lymph node involvement, histological type, histological grade, extent of residual disease and age at the time of diagnosis

When serum levels of CYFRA 21-1, taken before therapy, were grouped by CA 125 serum levels, tumour stage, lymph node involvement, histological type, histological grade, extent of residual disease and age at the time of diagnosis, we found no statistically significant associations with the investigated clinicopathological parameters.

### Preoperative CYFRA 21-1 serum levels as prognostic factors in ovarian cancer

As CYFRA 21-1 lacks a clearly defined cut-off value, a cut-off value of 9.4 µg $l^{-1}$ was selected according to the 0.75 quantile (upper quartile) of serum concentrations measured in the panel of ovarian cancer patients (Obermair et al. 1997). Using the product limit method of Kaplan and Meier, we calculated the probability of pretreatment CYFRA 21-1 serum levels to predict the overall survival. Elevated CYFRA 21-1 serum levels before therapy were associated with a poor overall and disease-free survival (log-rank test, $P = 0.02$, Figure 2; and log-rank test, $P = 0.005$, Figure 3, respectively). Patients with and without elevated CYFRA 21-1 serum levels did not show statistically significant differences regarding treatment modality, performance status, mean age of the patients and distribution of tumour stage.

### CYFRA 21-1 and CA 125 serum levels in benign conditions

Serum levels of CYFRA 21-1 and CA 125 in pelvic inflammatory disease, endometriosis, liver cirrhosis and inflammatory bowel disease are shown in Table 1. Compared with normal controls, median serum levels of CYFRA 21-1 were found to be significantly elevated in patients with liver cirrhosis (Mann–Whitney *U*-test, $P = 0.0001$), but not in patients with pelvic inflammatory disease, endometriosis and inflammatory bowel disease.

### DISCUSSION

Although cytokeratin tumour markers have been investigated in a wide variety of human malignancies, few data on cytokeratins in ovarian cancer exist.

In the present study, serum CYFRA 21-1 showed a sensitivity of 41% and a specificity of 95% in ovarian cancer patients. The ROC

**Figure 2** Kaplan–Meier analysis regarding overall survival of ovarian cancer patients with CYFRA 21-1 serum levels above the cut-off level (9.4 µg $l^{-1}$; $n = 9$) and CYFRA 21-1 serum levels below the cut-off level (9.4 µg $l^{-1}$, $n = 28$)

© Cancer Research Campaign 1998

**Figure 3** Kaplan–Meier analysis regarding disease-free survival of ovarian cancer patients with CYFRA 21-1 serum levels above the cut-off level (9.4 μg $l^{-1}$; $n = 9$) and CYFRA 21-1 serum levels below the cut-off level (9.4 μg $l^{-1}$; $n = 28$)

curve shows that CYFRA 21-1 is not suitable as a screening marker for ovarian cancer. Given the low prevalence of the disease (50/100 000), the CYFRA 21-1 test would yield only 1 in 245 women with a positive test actually having the disease.

With respect to differential diagnosis of adnexal masses, elevated CYFRA 21-1 serum levels showed a positive correlation with the risk of presenting with malignant disease. The higher the CYFRA 21-1 levels, the higher was the risk of carrying a malignant ovarian cyst. This points to the fact that cytokeratin release is a genuine part of ovarian cancer development. However, our study also shows that CYFRA 21-1 does not reveal additional diagnostic information in the presence of the established tumour marker CA 125. This documents that CYFRA 21-1 is not clinically useful as an additional discriminator between the absence or presence of malignancy in patients intended to undergo surgery for suspicious ovarian cysts.

It has to be stressed that all tumour marker results have to be interpreted with caution. Even CA 125, which is generally established as a specific tumour marker for ovarian cancer, has been reported to be elevated in various benign conditions as well as in other malignancies, e.g. endometriosis and endometrial cancer (Kramer et al. 1993; Rose et al. 1994). This is also true for cytokeratins, which have been reported to be elevated in a wide variety of human malignancies (Gion et al. 1994; Sliutz et al. 1995). This fact is based on the abundant expression of cytokeratins, which are found in all epithelia and epithelia-derived tissues. Thus, cytokeratins or cytokeratin fragments are expressed in large quantities in all proliferating epithelial tissues. Diseases of the liver have been reported to be associated with elevated serum levels of cytokeratins (Sabbatini et al. 1988). This is consistent with our findings of elevated CYFRA 21-1 serum levels in patients with liver cirrhosis. When interpreting elevated CYFRA 21-1 serum levels, it has to be taken into account that CYFRA 21-1 is not a specific ovarian cancer tumour marker and that the coincidence of other benign diseases or malignancies may lead to elevated serum levels and consequently to false-positive test results.

CK 19 has been described as an indicator of epithelial injuries in inflammatory diseases, e.g. parodontosis and chronic bronchitis (Ouhayoun et al. 1990; Nakamura et al. 1997). In the present study, CYFRA 21-1 was not increased in inflammatory bowel disease or pelvic inflammatory disease. This indicates that inflammation per se is not sufficient to cause elevated CK levels.

In the present study, we found no correlation between CYFRA 21-1 serum levels and tumour stage. This finding supports the assumption that serum levels of cytokeratins are not reflective of the tumour bulk but rather indicative of strongly proliferating tumours (Bormer et al. 1994).

Elevated serum levels of cytokeratins have been shown to provide prognostic information in several malignancies (Kainz et al. 1994; Carpelan-Holmstrom et al. 1996). In our study, we found that increased CYFRA 21-1 serum levels before therapy are predictive of the patients' outcome in ovarian cancer. Patients with elevated CYFRA 21-1 serum levels had a shortened overall and disease-free survival. Additional studies with an increased number of patients are justified to clarify further the prognostic value of CYFRA 21-1 in ovarian cancer patients.

**Table 1** Median serum levels of CYFRA 21-1 and CA 125 in patients with ovarian cancer, benign ovarian cysts, pelvic inflammatory disease, endometriosis, inflammatory bowel disease, liver cirrhosis and in healthy control subjects

| | No. | Median serum levels of CYFRA 21-1 μG $l^{-1}$ (range) | Median serum levels of CA 125 μ $l^{-1}$ (range) |
|---|---|---|---|
| Ovarian Cancer | 37 | 2.4 (0.9–54.9) | 293 (14.8–19619) |
| Benign ovarian cysts | 90 | 1.3 (0.4–20.3) | 17.3 (3.1–1340) |
| Pelvic inflammatory disease | 38 | 1.3 (0.4–3.4) | 18.8 (4.9–361) |
| Endometriosis | 10 | 1.0 (0.7–1.8) | 62.9 (9.4–374) |
| Inflammatory bowel disease | 10 | 2.1 (1.3–5.6) | 14.3 (4.6–40.6) |
| Liver cirrhosis | 20 | 4.7 (1.9–8.2) | 293 (12.3–8546) |
| Healthy controls | 40 | 1.9 (0.1–11) | – |

In conclusion, our data indicate that CYFRA 21-1 is suitable neither as a screening marker nor as an additional tool for differential diagnosis of adnexal masses. Regarding benign diseases, patients with liver cirrhosis show extremely high cytokeratin levels, whereas inflammatory conditions are not invariably associated with elevated serum levels of CYFRA 21-1. In ovarian cancer patients, serum levels of CYFRA 21-1 are not associated with clinicopathological parameters. The most promising result of this study is the prognostic value of preoperative CYFRA 21-1 serum levels regarding overall and disease-free survival. Considering these results, CYFRA 21-1, while obviously not suitable for screening or differential diagnosis of adnexal masses, could be useful as an additional prognostic factor in ovarian cancer patients.

### ACKNOWLEDGEMENT

We thank Mrs Schoenthal for expert technical assistance.

### REFERENCES

Bodenmüller H, Donie F, Kaufmann M and Banauch D (1994) The tumor markers TPA, TPS, TPAcyk, and CYFRA 21-1 react differently with the keratins 8, 18 and 19. *Int J Biol Markers* **9**: 70–74

Bormer O (1994) From tissue polypeptide antigen to specific cytokeratin assays. *Tumor Biol* **15**: 185–187

Bouwens L, Lu WG and Dekrijger R (1997) Proliferation and differentiation in the human fetal endocrine pancreas. *Diabetologia* **40**: 398–404

Campbell M and Machin D (1996) *Medical Statistics – a Commonsense Approach*. John Wiley & Sons: Chichester

Carpelan-Holmstrom M, Haglund C, Lundin J, Alfthan H, Stenmann UH and Roberts PJ (1996) Independent prognostic value of preoperative serum markers CA 242, specific tissue polypeptide antigen and human chorionic gonadotropin beta, but not of carcinoembryonic antigen or tissue polypeptide antigen in colorectal cancer. *Br J Cancer* **74**: 925–929

DeLong E, DeLong D and Clarke-Pearson D (1988) Comparing the areas under two or more correlated Receiver Operating Characteristic curves: a non-parametric approach. *Biometrics* **44**: 837–845

Gion M, Mione R and Becciolini A (1994) Relationship between cytosol TPS, TPA and cell proliferation. *Int J Biol Markers* **9**: 109–114

Goumas PD, Mastronikolis NS, Mastorakou AN, Vassilakos PJ and Nikiforidis GC (1997) Evaluation of TATI and CYFRA 21-1 in patients with head and neck squamous cell carcinoma. *J Otorhinolaryngol Related Spec* **59**: 106–114

Grem J (1997) The prognostic importance of tumor markers in adenocarcinomas of the gastrointestinal tract. *Curr Opin Oncol* **9**: 380–387

Hosmer D and Lemeshow S (1989) *Applied Logistic Regression*. Wiley: New York

Kainz C, Sliutz G, Mustafa G, Bieglmayer C, Kölbl H, Reinthaller A and Gitsch G (1995) Cytokeratin subunit 19 measured by CYFRA 21-1 assay in follow-up of cervical cancer. *Gynecol Oncol* **56**: 402–405

Kainz C, Steiner G, Gitsch G, Sliutz G, Mustafa G and Kölbl H (1994) Tissue polypeptide specific antigen (TPS) in der biochemischen tumordiagnostik. *Klin Labor* **40**: 999–1005

Kramer B, Gohagan J, Prorok P and Smart C (1993) A National Cancer Institute sponsored screening trial for prostatic, lung, colorectal, and ovarian cancers. *Cancer* **71**: 589–593

Mobus V, Gerharz CD, Press U, Moll R, Beck T, Mellin W, Pollow K, Knapstein PG and Kreienberg R (1992) Morphological, immunohistochemical and biochemical characterization of six newly established human ovarian carcinoma cell lines. *Int J Cancer* **52**: 76–84

Moll R, Franke WW, Schiller DL, Geiger B and Krepler R (1982) The catalog of human cytokeratins: patterns of expression in normal epithelia, tumours and cultured cells. *Cell* **31**: 11–24

Moll R, Levy R, Czernobilsky B, Hohlweg-Majert P, Dallenbach-Hellweg G and Franke W (1983) Cytokeratins of normal epithelia and some neoplasms of the genital tract. *Lab Invest* **49**: 599–610

Nakamura H, Abe S, Shibata Y, Yuki H, Suzuki H, Saito H, Sata M, Kato S and Tomoike H (1997) Elevated levels of cytokeratin 19 in the bronchoalveolar lavage fluid of patients with chronic airway inflammatory diseases: a specific marker for bronchial epithelial injury. *Am J Resp Crit Care Med* **155**: 1217–1221

Novaes M, Bendit I, Garicochea B and Delgiglio A (1997) Reverse transcriptase polymerase chain reaction analysis of cytokeratin 19 expression in the peripheral blood mononuclear cells of normal female blood donors. *J Clin Pathol Molecular Pathol* **50**: 209–211

Obermair A, Kucera E, Mayerhofer K, Speiser P, Seifert M, Czerwenka K, Kaider A, Leodolter S, Kainz C and Zeillinger R (1997) Vascular endothelial growth factor (VEGF) in human breast cancer: correlation with disease-free survival. *Int J Cancer* **74**: 455–458

Ouhayoun JP, Goffaux JC, Sawaf MH, Shabana AH, Collin C and Forest N (1990) Changes in cytokeratin expression in gingiva during inflammation. *J Periodontal Res* **25**: 283–292

Plebani M, Basso D, Del Favero G, Ferrara C, Megiatto T and Foger P (1993) Clinical utility of TPS, TPA and CA 19–9 measurement in pancreatic cancer. *Oncology* **50**: 436–440

Pujol JL, Grenier J, Daures JP, Daver A, Pujol H and Michel FB (1993) Serum fragment of cytokeratin subunit 19 measured by CYFRA 21-1 immunoradiometric assay as a marker of lung cancer. *Cancer Res* **53**: 61–66

Rose PG, Sommers RM, Reale FR, Hunter RE, Fournier L and Nelson BE (1994) Serial serum CA 125 measurements for evaluation of recurrence in patients with endometrial carcinoma. *Obstet Gynecol* **84**: 12–16

Sabbatini S, Monti M and Fini A (1988) Tissue polypeptide antigen (TPA) modifications in hepatic cirrhosis, aggressive chronic hepatitis, persistent chronic hepatitis, and in minimal pathology. *Int J Biol Markers* **3**: 127–128

Senga Y, Kimura G, Hattori T and Yoshida K (1996) Clinical evaluation of soluble cytokeratin 19 fragments (CYFRA 21-1) in serum and urine of patients with bladder cancer. *Urology* **48**: 703–710

Sliutz G, Tempfer C, Kainz C, Mustafa G, Gitsch G, Koelbl H and Biegelmayer C (1995) Tissue polypeptide specific antigen and cancer associated serum antigen in the follow-up of ovarian cancer. *Anticancer Res* **15**: 1127–1129

Sundstrom BE and Stigbrand T (1994) Cytokeratins and tissue polypeptide antigen. *Int J Biol Markers* **9**: 102–108

Yamamoto K, Oka M, Hayashi H, Tangoku A, Gondo T and Suzuki T (1997) Cyfra 21 1 is a useful marker for esophageal squamous cell carcinoma. *Cancer* **79**: 1647–1655

Yanagibashi T, Gorai I, Nakazawa T, Miyagi E, Hirahara F, Kitamura H and Minaguchi H (1997) Complexity of expression of the intermediate filaments of six new human ovarian cancer cell lines: new expression of cytokeratin 20. *Br J Cancer* **76**: 829–835

© Cancer Research Campaign 1998